# “What If Applicants Fake Their Responses?”: Modeling Faking and Response Styles in High-Stakes Assessments Using the Multidimensional Nominal Response Model

**DOI:** 10.1177/00131644241307560

**Published:** 2025-01-23

**Authors:** Timo Seitz, Maik Spengler, Thorsten Meiser

**Affiliations:** 1 University of Mannheim, Germany; 2 HR Diagnostics AG, Stuttgart, Germany

**Keywords:** faking, socially desirable responding, high-stakes assessment, multidimensional item response theory, nominal response model

## Abstract

Self-report personality tests used in high-stakes assessments hold the risk that test-takers engage in faking. In this article, we demonstrate an extension of the multidimensional nominal response model (MNRM) to account for the response bias of faking. The MNRM is a flexible item response theory (IRT) model that allows modeling response biases whose effect patterns vary between items. In a simulation, we found good parameter recovery of the model accounting for faking under different conditions as well as good performance of model selection criteria. Also, we modeled responses from *N* = 3,046 job applicants taking a personality test under real high-stakes conditions. We thereby specified item-specific effect patterns of faking by setting scoring weights to appropriate values that we collected in a pilot study. Results indicated that modeling faking significantly increased model fit over and above response styles and improved divergent validity, while the faking dimension exhibited relations to several covariates. Additionally, applying the model to a sample of job incumbents taking the test under low-stakes conditions, we found evidence that the model can effectively capture faking and adjust estimates of substantive trait scores for the assumed influence of faking. We end the article with a discussion of implications for psychological measurement in high-stakes assessment contexts.

To measure constructs such as personality traits, interests, or attitudes in psychological and educational measurement contexts, researchers and practitioners make use of self-report questionnaires. Test-takers are typically instructed to indicate how much they agree with several statements using a rating scale with graded response categories. Hereby, researchers and practitioners rely on test-takers’ ability and willingness to report their true traits and states, even if the questionnaire is employed in high-stakes contexts. Indeed, there is ample research showing that constructs measured via self-report rating scales consistently predict variables such as academic success (e.g., Poropat, 2009), job performance (e.g., Barrick & Mount, 1991; Ones et al., 2007), and job satisfaction (e.g., Judge et al., 2002). However, responses to rating scale items are not solely determined by the construct of interest (i.e., the substantive trait) but capture other sources of systematic variance. Consider, for instance, high-stakes contexts like personnel selection, where test-takers are motivated to achieve a certain assessment result. In such situations, test-takers can be particularly expected to respond in a way that enhances their impression in the respective context, that is, engage in faking (Paulhus, 2002).

In this article, we apply a recent parametrization of the multidimensional nominal response model (MNRM; Takane & de Leeuw, 1987; see Falk & Cai, 2016; Thissen & Cai, 2016) to account for the response bias of faking and show the utility of the approach for high-stakes personality assessments. The herein demonstrated model yields estimates of substantive trait scores that are adjusted for the assumed influence of faking and provides a measure of each test-taker’s faking degree in a given assessment context.

## Background: Response Biases in Rating Scale Measures

### Response Styles

According to the framework by Jackson and Messick (1958), response biases can be conceptually divided into response styles and response sets. Response styles represent tendencies of test-takers to prefer certain rating scale categories irrespective of item content (Baumgartner & Steenkamp, 2001; Van Vaerenbergh & Thomas, 2013). Examples of response styles are the tendency to choose the highest or lowest response category of a rating scale (extreme response style, ERS), the tendency to choose the midpoint of a rating scale (midscale response style, MRS), and the tendency to generally agree with statements (acquiescent response style, ARS; see Van Vaerenbergh & Thomas, 2013, for an overview).

Research suggests that response styles are interindividual difference variables that are stable over time (e.g., Weijters et al., 2010; Wetzel et al., 2016) and consistent across the assessment of different traits (e.g., Austin et al., 2006; Wetzel et al., 2013). From a methodological perspective, response styles can bias substantive research findings since they affect both univariate and multivariate distributions of rating scale data (Baumgartner & Steenkamp, 2001; Van Vaerenbergh & Thomas, 2013). Univariate distributions are affected in terms of inflated or deflated means and variances, whereas multivariate distributions are primarily affected in terms of biased, typically inflated covariances (e.g., Böckenholt & Meiser, 2017). Unless response styles are statistically accounted for, interindividual differences in response styles imply different expected values of item responses and scale scores for test-takers who truly have the same substantive trait level (e.g., Bolt & Johnson, 2009), leading to biased diagnostic inferences (e.g., Plieninger, 2017).

### Faking as a Form of Socially Desirable Responding (SDR)

As opposed to stable response styles, response sets are conceptualized as response biases that are inherent to situational characteristics of a specific assessment context (Jackson & Messick, 1958). A prominent example of response sets is socially desirable responding (SDR), which is defined as “the tendency to give overly positive self-descriptions” (Paulhus, 2002, p. 50). That is, SDR can be regarded as a distortion of responses such that social expectations are met. Since social standards depend on the situation in which test-takers respond to questionnaire items (e.g., Kuncel & Tellegen, 2009), SDR is not a genuine response style but a response set inherent to a given assessment context (see Ziegler, 2015). According to Paulhus (1984, 2002), SDR has a self-directed (*self-deception*) and an other-directed form (*impression management*). The other-directed form represents a deliberate distortion of responses and is commonly referred to as faking.

Faking can have numerous adverse effects on the psychometric properties of a test (Ziegler et al., 2011). For instance, faking leads to considerably elevated scores on scales that measure desirable traits (Birkeland et al., 2006; Viswesvaran & Ones, 1999), which causes heavily skewed score distributions and ceiling effects because the range of possible scores in a test with a Likert-type rating scale is limited. Also, given that test-takers differ in their propensity to edit responses according to situational demands (see Griffith & Converse, 2011; Griffith et al., 2007), faking leads to systematically biased rank orders of test-takers, altering selection decisions based on test scores (e.g., Mueller-Hanson et al., 2003). Like response styles, faking also constitutes an additional source of systematic variance, which leads to an inflation of intercorrelations between scales of a personality inventory (e.g., Ellingson et al., 1999; Klehe et al., 2012; Schmit & Ryan, 1993). That is, faking distorts construct validity in terms of divergent validity by inducing strong correlations between scales that should only exhibit weak relationships.

Besides effects on the psychometric properties of a test, SDR and faking can also be looked at from a substantive research perspective (see Bensch et al., 2019; Marcus, 2009; Ziegler, 2011). In this case, faking is not regarded as a pure nuisance variable but as a construct that has psychological meaning and can be integrated into the nomological network of interindividual difference variables. For instance, it is possible that people with certain personality characteristics are more inclined than others to engage in SDR and faking, or that SDR and faking are associated with certain levels of cognitive ability. Concerning personality, several studies found positive relationships between faking and the Big Five personality factors (see Li & Bagger, 2006, for a meta-analysis), even when the Big Five were assessed by observer ratings of personality (Ones et al., 1996) and when statistical modeling was used to account for faking (Brown & Böckenholt, 2022). Concerning cognitive ability, evidence has been mixed (e.g., Evans & Forbach, 1982; Schermer & Vernon, 2010). However, when faking is conceptualized as the tendency to create favorable scores in a high-stakes assessment, correlations between faking and cognitive ability are typically positive. Wetzel et al. (2021), for instance, reported a small positive correlation between faking in an experimental application situation and general intelligence.

### Previous Approaches to Accounting for Response Biases in Rating Scale Measures

#### Response Styles

Several approaches have been developed in recent decades to account for response styles (see Henninger & Meiser, 2020, 2022, for overviews). Early methods make use of descriptive statistics to quantify the extent to which test-takers engage in stylistic responding (e.g., number of extreme vs. nonextreme responses to quantify ERS; Bachman & O’Malley, 1984; Greenleaf, 1992). Other techniques apply mixture item response theory (IRT) models to identify latent subpopulations of test-takers differing in the use of response categories (e.g., Eid & Rauber, 2000; Meiser & Machunsky, 2008). A more recent approach treats response styles as continuous latent variables in multidimensional IRT models for ordinal data (Bolt & Newton, 2011; Falk & Cai, 2016; Henninger & Meiser, 2020). These models are special cases of the MNRM and incorporate response styles as additional latent dimensions along with substantive traits (see Method for Modeling Faking section for details). As Wetzel and Carstensen (2017) demonstrated, such modeling of response styles along with the Big Five personality factors considerably increases model fit and leads to adjusted estimates of substantive trait scores, particularly if test-takers have pronounced response style levels (see also Bolt & Johnson, 2009; Falk & Ju, 2020).

#### SDR and Faking

SDR and faking have been studied by psychologists and survey methodologists for more than half a century, resulting in several different approaches to account for it. One prominent technique has been to measure self-deception and impression management using designated SDR scales (see Paulhus, 2002; Paulhus & Trapnell, 2008, for overviews). In general, SDR scales consist of items capturing desirable behaviors that are hardly shown by anybody (e.g., always picking up other people’s liter on the street) or, vice versa, items capturing undesirable behaviors that are actually very common (e.g., occasionally driving faster than the speed limit). A test-taker who endorses many of the former and few of the latter items would receive a high score on an SDR scale. A striking limitation of SDR scales, however, is that they are confounded with substantive trait variance (e.g., de Vries et al., 2014; McCrae & Costa, 1983). This is reflected in the typical finding of moderate to strong correlations between SDR scales and the Big Five (Li & Bagger, 2006; Ones et al., 1996). That is, SDR scales measure, at least to a certain extent, substantive personality traits as opposed to only response bias (e.g., McCrae & Costa, 1983; Uziel, 2010). To adjust test-takers’ substantive trait scores for SDR, it is hence not appropriate to use residuals from a regression of substantive trait scale scores on SDR scale scores, because this removes a considerable proportion of substantive variance from test-takers’ trait scores (Griffith & Peterson, 2008; Reeder & Ryan, 2011).

Besides SDR scales, other methods to quantify faking have been proposed, such as overclaiming techniques (Paulhus et al., 2003), exploratory mixture models to identify latent faking classes (e.g., Zickar et al., 2004), or person-fit indices in IRT models (e.g., LaHuis & Copeland, 2009; Zickar & Drasgow, 1996). However, even if these measures were effective in capturing faking in terms of a genuine response bias, they primarily provide an additional piece of information regarding individual test-takers and do not necessarily yield faking-adjusted substantive trait estimates for all test-takers.

Instead, to capture faking and at the same time get faking-adjusted estimates of substantive trait scores, latent variable modeling can be used. Such models simultaneously consider the influence of substantive traits *and* faking on item responses and thus take faking directly into account when estimating model parameters. This can afford substantive trait score estimates that are more adequately adjusted for faking, Also, this yields model-based estimates of each test-taker’s faking degree, which can shed light on the substantive nature of faking by facilitating the examination of correlations between faking and other psychological constructs.^1^

## Method for Modeling Faking

### The Multidimensional Nominal Response Model (MNRM)

Building on recent advancements in IRT response style modeling (see Falk & Cai, 2016), this article demonstrates an extension of the MNRM to account for faking along with substantive traits and response styles. The MNRM was originally proposed by Takane and de Leeuw (1987) as a multidimensional generalization of Bock’s (1972) approach to modeling nominal (i.e., categorical) item responses with a single latent dimension representing the trait of interest. In the multidimensional extension, the probability that test-taker *n* chooses response category *k* out of a set of *K*+1 categories on item *i* is modeled with a multinomial logistic function in which multiple latent dimensions are assumed to influence item responses:

(1) $$p(Y_{\mathrm{ni}}\;=\;k\;|\;\theta_{n},\gamma_{i},\alpha_{i},S_{i})=\frac{\exp((\alpha_{i}{}^{\circ}s_{\mathrm{ik}})'\theta_{n}+\gamma_{\mathrm{ik}})}{\sum_{m=0}^{K}\exp((\alpha_{i}{}^{\circ}s_{\mathrm{im}})'\theta_{n}+\gamma_{\mathrm{im}})}$$

$$\mathrm{with}\;\theta_{n}=\left(\begin{array}{c} \theta_{n1}\\ \vdots \\ \theta_{\mathrm{nd}}\\ \vdots \\ \theta_{\mathrm{nD}} \end{array}\right),\gamma_{i}=\left(\begin{array}{ccccc} \gamma_{i0} & \cdots & \gamma_{\mathrm{ik}} & \cdots & \gamma_{\mathrm{iK}} \end{array}\right),$$

$$\alpha_{i}=\left(\begin{array}{c} \alpha_{i1}\\ \vdots \\ \alpha_{\mathrm{id}}\\ \vdots \\ \alpha_{\mathrm{iD}} \end{array}\right),\;\mathrm{and}\;S_{i}=\left(\begin{array}{ccccc} s_{i10} & \cdots & s_{i1k} & \cdots & s_{i1K}\\ \vdots & & \vdots & & \vdots \\ s_{\mathrm{id}0} & \cdots & s_{\mathrm{idk}} & \cdots & s_{\mathrm{idK}}\\ \vdots & & \vdots & & \vdots \\ s_{\mathrm{iD}0} & \cdots & s_{\mathrm{iDk}} & \cdots & s_{\mathrm{iDK}} \end{array}\right),$$

where *Y_ni_* is a discrete random variable that represents the observed item response of test-taker *n* on item *i* (*Y_ni_*∈ {0, 1, . . ., *k, . . ., K*}), *k* denotes its realization, ***θ***_*n*_ is a vector of test-taker *n*’s levels on the *D* dimensions, and ***γ***_*i*_ is a vector of item- and category-specific intercepts. The parameterization in Equation 1 (Falk & Cai, 2016; Thissen & Cai, 2016) also incorporates item-specific slopes *α*_*id*_ that reflect the relation between item *i* and dimension *d* (collected in vector ***α***_*i*_), and separates them from item- and category-specific scoring weights *s*_*idk*_ that reflect the relation between dimension *d* and category *k* on item *i* (collected in matrix ***S***_*i*_). Vector ***α***_*i*_ and column vector ***s***_*ik*_ from matrix ***S***_*i*_ are linked through the Hadamard product (denoted by the symbol °), such that parameters pertaining to the same dimension *d* are multiplied before the resulting vector is transposed and multiplied by vector ***θ***_*n*_. This leads to a sum of products *α*_*id*_*s*_*idk*_*θ*_*nd*_ over the *D* dimensions. After *γ*_*ik*_ is added to this sum, the resulting term is transformed through a multinomial logistic function to a range from 0 to 1 to yield the model-implied probability of an item response. Table 1 provides an overview of parameters in the MNRM.

**Table 1.** Overview of Parameters in the Multidimensional Nominal Response Model (MNRM).

Parameter	Symbol	Estimated or fixed	Meaning
Item slope	*α*_*id*_	Estimated	Value reflecting the relation between item *i* and dimension *d* (aka item discrimination); all freely estimated if model is identified by fixing variances of latent dimensions
Scoring weight	*s*_*idk*_	Fixed	Value reflecting the relation between dimension *d* and category *k* on item *i*; fixed to theoretical values
Item-category intercept	*γ*_*ik*_	Estimated (*γ*_*i*0_ fixed to 0)	Value reflecting an additive constant of item *i* and category *k* (related to item-category thresholds and item difficulty); *γ*_*i*0_ fixed for model identification
Latent mean	*μ*_*d*_	Fixed	Expected value of latent dimension *d*; fixed for model identification
Latent covariance	*ρ*_*dd′*_	Estimated (*ρ*_*dd*_ fixed to 1)	Covariance between latent dimensions *d* and *d′* (correlation if variances of latent dimensions are 1)
Person parameter	*θ*_*nd*_	Estimated	Value reflecting person *n*’s level on dimension *d* (aka trait scores / trait estimates)

*Note.* This overview of parameters in the multidimensional nominal response model (MNRM) applies to the use of the model as in the present article. Other parametrizations and identification constraints are possible (see Falk & Cai, 2016). Regarding estimation, item parameters and latent correlations are estimated in a first step, whereas person parameters are estimated in a second step treating the other parameters as fixed.

To estimate the model, certain identification constraints need to be imposed (see Falk & Cai, 2016; Henninger & Meiser, 2020; Johnson & Bolt, 2010, for details). Assuming that the *D* latent dimensions are multivariate normally distributed with expectation vector µ and variance-covariance matrix Σ, a typical restriction is to fix the expectations of all latent dimensions to 0 and their variances to 1. Also, the intercept of the first category is usually fixed to 0 for all items. Furthermore, because scoring weights reflect the relation between a dimension and a category on a given item, scoring weights can be specified a priori if one has theoretical assumptions about relations between dimensions and categories. For items with ordinal categories, scoring weights of a dimension representing a substantive trait are typically set to equally spaced values. In the case of a 7-point Likert scale, the scoring weight vector $\left(\begin{array}{ccccccc} 0 & 1 & 2 & 3 & 4 & 5 & 6 \end{array}\right)$ can be specified to reflect the assumption that higher substantive trait levels trigger the selection of higher response categories. Since this assumption applies to all items designed to measure a certain substantive trait, the same scoring weight vector is specified for every item pertaining to the respective substantive trait. To account for tendencies of test-takers toward response categories that are independent of item content, response style dimensions can be added to the model. For these dimensions, scoring weights can be specified according to the definition of a particular response style. For ERS on a 7-point Likert scale, one can set a scoring weight vector of $\left(\begin{array}{ccccccc} 1 & 0 & 0 & 0 & 0 & 0 & 1 \end{array}\right)$ to reflect the assumption that higher ERS levels increase the probability of choosing extreme response categories. For MRS, one can set scoring weights to $\left(\begin{array}{ccccccc} 0 & 0 & 0 & 1 & 0 & 0 & 0 \end{array}\right)$ , reflecting the assumption that higher MRS levels make midpoint responses more likely. Because response styles are conceptualized to be independent of item content, the same scoring weight vector is specified for every item of the questionnaire.

### Application to Faking

Considering that scoring weights reflect the relation between latent dimensions and response categories, it is straightforward to apply this logic to faking and model it along with substantive traits and response styles. To empirically determine scoring weights for the faking dimension, one can assess the relation between social desirability and response categories by letting a sample of participants rate each response category of each questionnaire item regarding desirability with respect to a particular assessment context. Using such a procedure, Kuncel and Tellegen (2009) found that the relationship between response categories and desirability largely varies between items and is often not strictly monotonic. That is, higher response categories can be associated with higher desirability for some items (e.g., “I am well-organized”), whereas response categories in the middle range of the rating scale can have highest desirability for other items (e.g., “I am talkative”). Hence, if item- and category-specific desirability ratings are used as scoring weights, scoring weight vectors of faking are neither constant across items nor globally redundant to scoring weight vectors of substantive trait dimensions.

Consider a situation where responses to a questionnaire designed to measure five substantive traits with a 7-point Likert scale are modeled such that effects of substantive traits, response styles (e.g., ERS and MRS), and faking are accounted for. The scoring weight matrix ***S***_*i*_ for items measuring the first substantive trait can be written as

(2) $$S_{i}=\left(\begin{array}{ccccccc} 0 & 1 & 2 & 3 & 4 & 5 & 6\\ 0 & 0 & 0 & 0 & 0 & 0 & 0\\ 0 & 0 & 0 & 0 & 0 & 0 & 0\\ 0 & 0 & 0 & 0 & 0 & 0 & 0\\ 0 & 0 & 0 & 0 & 0 & 0 & 0\\ 1 & 0 & 0 & 0 & 0 & 0 & 1\\ 0 & 0 & 0 & 1 & 0 & 0 & 0\\ s_{i\mathrm{Faking}0} & s_{i\mathrm{Faking}1} & s_{i\mathrm{Faking}2} & s_{i\mathrm{Faking}3} & s_{i\mathrm{Faking}4} & s_{i\mathrm{Faking}5} & s_{i\mathrm{Faking}6} \end{array}\right).$$

The first five rows pertain to the five substantive traits, the sixth and seventh row to ERS and MRS, and the eighth row to faking. Because item *i* is designed to measure only the first of the substantive traits, scoring weights of the second to fifth substantive trait dimensions are set to 0. For the faking dimension, item *i*’s desirability ratings can be plugged in as scoring weights of faking to reflect item-specific desirability characteristics. Thus, the model simultaneously accounts for response styles, whose effect patterns are assumed to be invariant across items, and faking, whose effect patterns are assumed to be specific to individual items. Because this separates response styles from the response set of faking, the application of the MNRM to faking constitutes an important extension over traditional approaches to modeling response tendencies (see Discussion section for specific advantages over recent faking models).^2^

## Simulation

To evaluate the described model with respect to its ability to recover focal model parameters better than models not accounting for faking, we conducted a simulation analysis using the *mirt* package (Chalmers, 2012) in the *R* environment (version 4.2.1). Along with the examination of parameter recovery, the simulation also had the purpose of investigating how well model selection criteria can correctly identify the underlying population model.

### Data Generation and Fitted Models

In the simulation, we varied the presence of faking in item responses (not present vs. present) as well as the sample size of simulated test-takers per dataset (250 vs. 500 vs. 1,000 vs. 3,000). Concerning the selection of sample size conditions, we oriented on minimum sample size requirements for polytomous IRT models (Dai et al., 2021) as a lower bound, on sample size recommendations for complex multidimensional IRT models and typical sample sizes in the psychometric literature (de Ayala, 2022), as well as on the sample size of the dataset in our empirical demonstration as an upper bound. Irrespective of the simulation condition, we simulated a situation in which five substantive traits were measured by 10 items respectively on a 7-point Likert scale. Since rating scale measures are usually contaminated with response styles (e.g., Bolt & Newton, 2011; Wetzel & Carstensen, 2017), we included ERS and MRS in the generation of item responses. Specifically, we proceeded as follows to generate the data (the entire simulation syntax can be found at https://osf.io/f8vgp/):

1. Item slope parameters *α*_*id*_: Slopes of substantive trait, ERS, and MRS dimensions were drawn from *U*(min = 0.25, max = 0.75). In conditions in which faking was present, slopes of faking were also sampled from *U*(min = 0.25, max = 0.75), implying that all dimensions had on average an equivalent impact on item responses in these conditions. In conditions in which faking was not present, faking slopes were set to 0 such that the faking dimension could not influence the generated item responses in these conditions.
2. Scoring weights *s*_*idk*_: Scoring weights of substantive traits and response styles were set to values as described above (see Equation 2). Scoring weights of faking were item-specific to emulate a situation in which the relation between response categories and desirability varies between items. In particular, within each substantive trait scale, scoring weight vectors of faking were generated to simulate relationships between categories and desirability that were monotonically increasing, nonmonotonically increasing, or inverted-U-shaped (cf. Figure 3 and the simulation syntax for details).^3^
3. Item-category intercept parameters *γ*_*ik*_: For all items, the intercept of the first category was fixed to 0. The other intercepts were generated by drawing item- and category-specific threshold values τ_*ik*_ from *MVN*(µ = $\bar{\tau }$ , Σ = ***T***), where $\bar{\tau }$ = (−1.5 −0.9 −0.3 0.3 0.9 1.5)' and $T=\mathrm{diag}\left(\begin{array}{cccccc} 0.7 & 0.7 & 0.7 & 0.7 & 0.7 & 0.7 \end{array}\right)$ ,^4^ and transforming them to cumulative thresholds that represent intercepts: $\gamma_{\mathrm{ik}}=-\sum_{m=0}^{k}\tau_{\mathrm{im}}$ .
4. Person parameters *θ*_*nd*_: Depending on the sample size condition, person parameters of *N* simulated test-takers were drawn from *MVN*(µ, Σ). µ was set to $\left(\begin{array}{cccccccc} 0 & 0 & 0 & 0 & 0 & 0 & 0 & 0 \end{array}\right)$ , and latent variances in Σ were fixed to 1 for all dimensions. Latent correlations between substantive traits were set to values representing DeYoung’s (2006) findings on latent correlations between the Big Five. ERS and MRS were set orthogonal to each other and to all other dimensions. Latent correlations of faking with the five substantive traits were set to .00, .10, −.10, .30, and −.30.
5. The generated item and person parameters were used to simulate item responses based on the multinomial logistic function in Equation 1.
6. Steps 1 to 5 were repeated such that 1,000 datasets were simulated per condition.

All steps were carried out using the R packages *mirt*, *MASS* (Venables & Ripley, 2002), and *SimDesign* (Chalmers & Adkins, 2020). To all 1,000 simulated datasets per condition, four models were fitted: a model only accounting for the five substantive traits, a model accounting for substantive traits and ERS, a model accounting for substantive traits, ERS, and MRS, and a model accounting for substantive traits, ERS, MRS, and faking. Typical constraints were imposed for model identification, that is, expectations of all latent dimensions were fixed to 0, variances to 1, and the intercept of the first category to 0 for all items. Scoring weights of latent dimensions were specified as in the data generation. Because of the models’ high dimensionality, the Metropolis-Hastings Robbins-Monro (MH-RM) algorithm (Cai, 2010) as implemented in the *mirt* package was used to estimate the models. The MH-RM algorithm is a Bayesian estimation procedure that combines elements from Markov chain Monte Carlo (MCMC) methods with stochastic approximation techniques and converges to the maximum likelihood solution. To estimate person parameters in the high-dimensional models, maximum a-posteriori (MAP) scores were computed (see Embretson & Reise, 2000).

### Results of the Simulation

To examine the performance of model selection criteria, we considered the proportions with which different model selection criteria (namely, the likelihood-ratio (LR) test with a significance level of *α* = .05, the Akaike information criterion (AIC), and the Bayesian information criterion (BIC)) correctly identified the underlying population model across replications in each condition. Because ERS and MRS were part of the data-generating process in all conditions, the model accounting for substantive traits, ERS, and MRS represented the population model in conditions in which faking was not present in the data, whereas the model additionally accounting for faking represented the population model when faking was present. As can be seen in Table 2, in conditions in which faking was not present, all model selection criteria performed well at correctly identifying the model including substantive traits and both response styles as the population model. The LR test comparing the model including substantive traits, ERS, and MRS with the model additionally including faking selected the model without faking in 94.7% to 97.6%, which implies type I error rates close to the nominal significance level. AIC and BIC chose the model without faking even in 95.1% to 99.2% and 99.0% to 100.0%, respectively. In conditions in which faking was present, the LR test as well as AIC and BIC correctly selected the model including faking in all replications. That is, even in smaller samples, the empirical power for identifying the model including faking as the population model was 100% for all three model selection criteria.

**Table 2.** Simulation: Proportions of Correctly Identified Population Models.

Sample size condition	Faking condition	
	Faking not present	Faking present
***LR test*** (α = ***.05):***		
250	95.9%	100.0%
500	97.2%	100.0%
1,000	97.6%	100.0%
3,000	94.7%	100.0%
**AIC**		
250	97.1%	100.0%
500	99.2%	100.0%
1,000	98.6%	100.0%
3,000	95.1%	100.0%
***BIC:***		
250	99.0%	100.0%
500	100.0%	100.0%
1,000	100.0%	100.0%
3,000	99.2%	100.0%
		

*Note.* Proportions are based on 1,000 replications per condition. In simulation conditions in which faking was not present in the data generation, a model accounting for substantive traits, extreme response style (ERS), and midscale response style (MRS) was the underlying population model, whereas a model additionally accounting for faking was the population model in conditions in which faking was present. LR test = likelihood-ratio test; AIC = Akaike information criterion; BIC = Bayesian information criterion.

**Figure 1.**
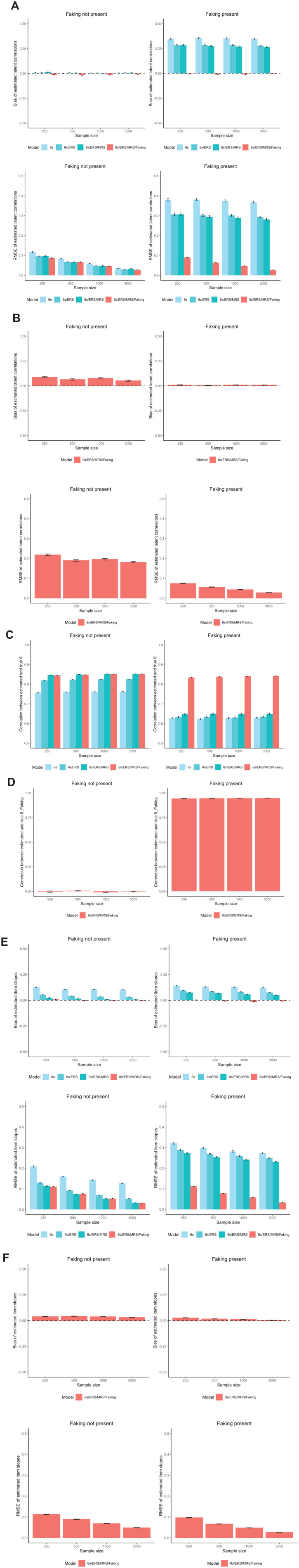
Simulation: Parameter Recovery of Focal Model Parameters. (A) Latent Correlations Between Substantive Traits; (B) Latent Correlations Between Faking and Substantive Traits; (C) Person Parameters of Substantive Traits; (D) Person Parameters of Faking; (E) Item Slopes of Substantive Traits; and (F) Item Slopes of Faking. *Note.* Results for parameters related to substantive traits are aggregated across the five substantive traits used in the simulation. Values of parameter recovery reflect the mean bias, the root mean square error (RMSE), or the mean correlation (using Fisher’s z-transformation) between estimated and true parameter values across replications within a condition. Error bars represent the standard error of the mean. θs = only substantive traits modeled; θs/ERS = substantive traits and ERS modeled; θs/ERS/MRS = substantive traits, ERS, and MRS modeled; θs/ERS/MRS/Faking = substantive traits, ERS, MRS, and faking modeled.

To evaluate the recovery of focal model parameters (namely, latent correlations, person parameters, and item slopes), we looked at bias to examine if parameters were systematically over- or underestimated as well as at root mean square error (RMSE) to examine estimation precision. For the recovery of person parameters, we considered the correlation between estimated and true parameters. Results are displayed in Figure 1. Regarding latent correlations between substantive traits (see Figure 1A), models without and with faking dimension yielded essentially unbiased estimates in conditions in which faking was not present. As can be expected, RMSE reduced in larger samples and in models accounting for response styles. In conditions in which faking was present, however, models without faking dimension yielded largely positively biased estimates of latent correlations between substantive traits. Accounting for response styles only slightly attenuated this bias. Also, RMSE did not reduce with larger sample size in these models. Crucially, adding a faking dimension eliminated the bias and drastically reduced RMSE, particularly in larger samples. Concerning latent correlations between faking and substantive traits (see Figure 1B), parameters could be recovered without bias and with smaller RMSE in larger samples when faking was present in the data. When faking was not part of the data generation and faking was nonetheless modeled, a small positive bias occurred. That is, instead of estimating zero correlations, the model on average estimated small positive latent correlations between faking and substantive traits even though faking was absent in the data. As expected, RMSE was more pronounced in smaller samples.

Regarding the estimation of person parameters of substantive traits (see Figure 1C), recovery improved in all conditions when accounting for both ERS and MRS along with substantive traits. When faking was not present in the data, additionally accounting for faking did not change parameter recovery. However, when faking was present, recovery was considerably better in models accounting for faking than in models ignoring faking. With respect to person parameters of faking (see Figure 1D), parameters could unsurprisingly not be estimated properly in conditions in which faking was not present in the data. In conditions in which faking was present, however, faking person parameters could be recovered precisely. Person parameter recovery was independent of sample size in all conditions.

Concerning item slopes of substantive traits (see Figure 1E), parameters were positively biased in models that lacked dimensions which were part of the data generation. In conditions in which faking was not present, item slopes were biased in the model only accounting for substantive traits as well as in the model accounting for substantive traits and ERS, whereas they were unbiased in the model accounting for substantive traits, ERS, and MRS as well as in the model additionally accounting for faking. RMSE was most pronounced in the model only accounting for substantive traits, and reduced when ERS and MRS were accounted for and when the sample size was larger. In conditions in which faking was present, item slopes of substantive traits were positively biased and had pronounced RMSE in the models not including faking. Only when models accounted for faking, estimates were unbiased and RMSE considerably reduced, especially in larger samples. Item slopes of faking (see Figure 1F) were marginally positively biased in smaller samples when faking was present. However, this bias was eliminated in larger samples. When faking was not present, similar to the estimation of latent correlations between faking and substantive trait, item slopes of faking consistently had a small positive bias, that is, they were on average estimated a bit larger than 0 despite the absence of faking in the data. Again, RMSE reduced in larger samples.

## Empirical Demonstration

The findings of the simulation suggest it is worthwhile to account for faking in rating scale data using the MNRM, especially if responses are indeed contaminated with faking. To demonstrate the faking modeling approach in empirical high-stakes assessment data, we modeled a dataset from a job application context. The empirical demonstration should address three research questions:

Research Question 1: Does modeling faking significantly increase model fit?
Research Question 2: Does the faking dimension adjust (a) inflated correlations between substantive traits and (b) inflated means?
Research Question 3: How is faking related to other psychological constructs?

A more detailed presentation of the empirical analyses can be found in Online Supplement I at https://osf.io/f8vgp/.

### Datasets

The data for the empirical demonstration came from a Germany-based testing company that develops psychological assessment tools for personnel selection. The dataset contained data from *N* = 3,046 job applicants who had taken a Big Five personality test (48 items, 7-point Likert type scale) and several cognitive ability tests as part of their application for an apprenticeship at a German organization in the financial industry. For eventually hired applicants (*N* = 546), demographic variables were available. In this subsample, 60.4% were female (39.6% male), and the mean age was *M* = 18.22 years (*SD* = 1.98, *range* = [14, 29]). All models were fitted to the sample of *N* = 3,046 job applicants (high-stakes condition). In addition, data from *N* = 365 job incumbents (i.e., employed apprentices at the time of data collection) were made available (low-stakes condition), which we used for validation of the model in Research Question 2. These data had been collected as part of an evaluation study of the test battery. In this sample, 57.3% of job incumbents were female (42.7% male), with a mean age of *M* = 20.90 years (*SD* = 2.06, *range* = [17, 33]).

### Pilot Study

**Figure 2.**
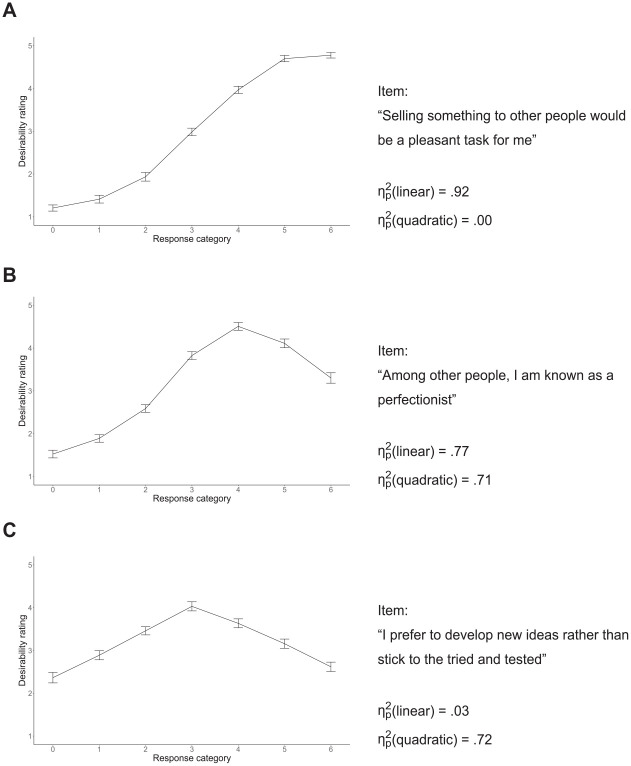
Empirical Demonstration: Desirability Trajectories of Three Exemplary Items. (A) Only Linear Trend Significant at *α* = .001; (B) Linear and Quadratic Trend Significant at *α* = .001; and (C) Only Quadratic Trend Significant at *α* = .001. *Note.* Mean desirability ratings are based on *N* = 63 participants. Error bars represent the standard error of the mean. $\eta_{p}^{2}$ values are partial proportions of variance explained by the linear and quadratic trend, respectively.

To determine scoring weights for the faking dimension, we ran a pilot study in which participants rated the social desirability of every response category for every item of the Big Five questionnaire used in the actual assessment (cf. Kuncel & Tellegen, 2009; all materials are available on the Open Science Framework). Therefore, we instructed participants to take the perspective of a high school graduate currently applying for an apprenticeship at a financial institution (i.e., a bank) and rate desirability with respect to this context. Figure 2 shows the resulting desirability values for three exemplary items.

### Results of the Empirical Demonstration

**Figure 3.**
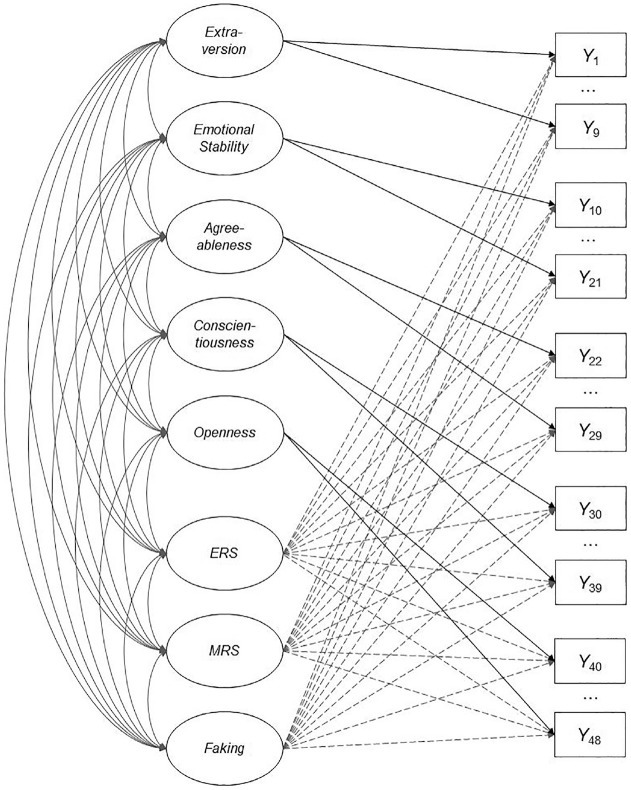
Dimensional Structure of the Full Model in the Empirical Demonstration. *Note.* The same dimensional structure also applies to the full model in the simulation, however, with a different number of items. ERS = extreme response style; MRS = midscale response style.

Like in the simulation, we used R for data preparation, model estimation, and subsequent analyses, in particular the *mirt* package to specify and estimate the respective IRT models with the MH-RM algorithm. We imposed the same identification constraints as described above, and specified scoring weights as in Equation 2. For scoring weights of the faking dimension, we used the mean desirability ratings from the pilot study, which we linearly transformed to a range from 0 to 1 to achieve a comparable scoring weight metric of response bias dimensions. We fitted the same four models as in the simulation, both with equality-constrained item slopes within dimensions and with unconstrained item slopes. Figure 3 depicts a graphical illustration of the full model. For all analyses, we set a significance level of *α* = .001.

#### Model Fit (Research Question 1)

All models converged within less than 339 MH-RM iterations. Table 3 provides an overview of estimated parameters and model fit. Irrespective of constraining slopes within dimensions, the stepwise addition of ERS and MRS to the Big Five consistently led to a significantly increased model fit according to the LR test. Crucially, the addition of faking increased model fit further. The same conclusions could be drawn when considering AIC and BIC as well as absolute fit indices such as the root mean square error of approximation (RMSEA; Maydeu-Olivares & Joe, 2014) and the Tucker-Lewis index (Cai & Monroe, 2013). Comparisons of models with equality-constrained versus unconstrained slopes indicated that setting slopes free significantly increased fit for all models, χ^2^s > 3,326.9, *p*s < .001. Correspondingly, the full model with unconstrained slopes was used for further analyses. The mean item slope of faking in this model was $\bar{\alpha }$ ._Faking_ = 1.69 (see Table S.I.2 in Online Supplement for all item parameter estimates in this model).

**Table 3.** Empirical Demonstration: Overview of Estimated Parameters and Model Fit Measures.

		Estimated parameters				Model fit measures						
Model		Total number	Slopes	Intercepts	Covariances	*C*_2_ *(df*), *p*-value	RMSEA	TLI	Log-likelihood	AIC	BIC	LR test
Equality-constrained slopes within dimensions	B5	303	5	288	10	13,204.6 (1,113), < .001	.060	.878	−210,716.0	422,037.9	423,862.5	
	B5/ERS	309	6	288	15	13,485.9 (1,107), < .001	.061	.875	−204,202.1	409,022.2	410,882.8	*X^2^*(6) = 13,027.8, *p* < .001
	B5/ERS/MRS	316	7	288	21	12,530.3 (1,100), < .001	.058	.884	−203,739.2	408,110.5	410,013.3	*X^2^*(7) = 925.7, *p* < .001
	B5/ERS/MRS/Faking	324	8	288	28	11,633.6 (1,092), < .001	.056	.892	−202,499.3	405,646.5	407,597.5	*X^2^*(8) = 2,480.0, *p* < .001
Unconstrained slopes	B5	346	48	288	10	11,835.0 (1,070), < .001	.057	.887	−207,550.9	415,793.8	417,877.3	
	B5/ERS	399	96	288	15	9,654.0 (1,017), < .001	.053	.905	−202,116.2	405,030.4	407,433.1	*X^2^*(53) = 10,869.4, *p* < .001
	B5/ERS/MRS	453	144	288	21	9,178.6 (963), < .001	.053	.905	−201,612.1	404,130.2	406,858.0	*X^2^*(54) = 1,008.2, *p* < .001
	**B5/ERS/MRS/Faking**	**508**	**192**	**288**	**28**	**7,224.5 (908), < .001**	**.048**	**.922**	−**200,835.8**	**402,687.6**	**405,746.6**	***X^2^*(55) = 1,552.7, *p* < .001**

*Note.* Models were fitted to responses from *N =* 3,046 test-takers on I = 48 items with *K*+1 *=* seven response categories. Expectations and variances of latent dimensions were fixed to 0 and 1, respectively, in all models. Scoring weights were specified a priori. *C*_2_ = limited information fit statistic *C*_2_ (Cai & Monroe, 2014); RMSEA = root mean square error of approximation; TLI = Tucker-Lewis index; AIC = Akaike information criterion; BIC = Bayesian information criterion; LR test = likelihood-ratio test (here: hierarchical comparison of nested models); B5 = Big Five; ERS = extreme response style; MRS = midscale response style. The best fitting model is printed in bold.

#### Validation of the Faking Modeling Approach (Research Question 2)

##### Latent Correlations

To validate the faking modeling approach, we first examined latent correlations between substantive traits (see Table 4). In the model including only the Big Five, estimated latent correlations were very high. When accounting for ERS and MRS, latent correlations decreased slightly but were still higher than typical low-stakes findings on Big Five intercorrelations. Once faking was added to the model, however, latent correlations reduced to more plausible levels.

**Table 4.** Empirical Demonstration: Estimated Latent Correlations.

(a) Model: B5								
	E	ES	A	C	O			
E	1							
ES	.58	1						
A	.62	.81	1					
C	.63	.60	.67	1				
O	.76	.52	.58	.70	1			
(b) Model: B5/ERS								
	E	ES	A	C	O	ERS		
E	1							
ES	.44	1						
A	.36	.75	1					
C	.42	.41	.42	1				
O	.60	.29	.28	.5	1			
ERS	.05	−.07	−.04	.13	.08	1		
(c) Model: B5/ERS/MRS								
	E	ES	A	C	O	ERS	MRS	
E	1							
ES	.44	1						
A	.37	.75	1					
C	.42	.41	.42	1				
O	.61	.33	.31	.53	1			
ERS	.08	−.05	−.03	.16	.11	1		
MRS	.08	.11	.12	.11	.07	.29	1	
(d) Model: B5/ERS/MRS/Faking								
	E	ES	A	C	O	ERS	MRS	Faking
E	1							
ES	.20	1						
A	−.00	.48	1					
C	.26	.06	.10	1				
O	.46	−.02	−.02	.35	1			
ERS	.17	.06	.16	.31	.15	1		
MRS	.04	−.01	.02	.03	.02	.27	1	
Faking	.27	.26	.58	.31	.28	.11	−.01	1

*Note. N* = 3,046. All standard errors of latent correlations across models were smaller than 0.05. Slopes were unconstrained. B5 = Big Five; E = Extraversion; ES = Emotional Stability; A = Agreeableness; C = Conscientiousness; O = Openness; ERS = extreme response style; MRS = midscale response style.

##### Person Parameters in the High-Stakes Versus Low-Stakes Condition

**Figure 4.**
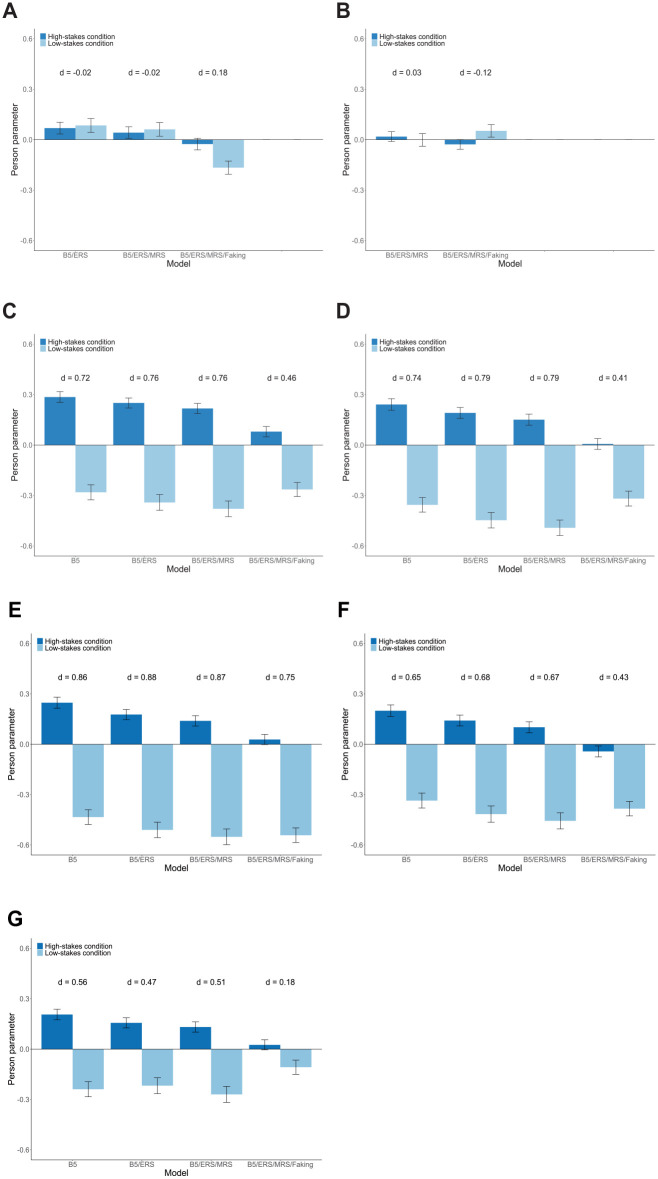
Empirical Demonstration: Mean Estimated Person Parameters of Response Styles and the Big Five for the High-Stakes and Low-Stakes Condition. (A) ERS; (B) MRS; (C) Extraversion; (D) Emotional Stability; (E) Agreeableness; (F) Conscientiousness; and (G) Openness. *Note.* *N* = 546 in the high-stakes condition; *N* = 365 in the low-stakes condition. Person parameters are maximum a-posteriori (MAP) scores. Error bars represent the standard error of the mean. In Figure 4A and 4B, none of the between-condition mean differences is significant at *α* = .001. In Figure 4C–4F, all between-condition mean differences are significant. In Figure 4G, all between-condition mean differences except the one in the B5/ERS/MRS/Faking model are significant. B5 = Big Five; ERS = extreme response style; MRS = midscale response style.

Next, we compared person parameters between the high-stakes and low-stakes condition. Therefore, we applied the models fitted to the responses from job applicants to the data from job incumbents. That is, to estimate person parameters (MAP scores) for test-takers in the low-stakes condition, we used the estimated model parameters from the models fitted to the high-stakes condition. This procedure should create a common scale of person parameters in both conditions (see Wetzel et al., 2021, who followed a similar approach). To limit the threat of confounds between the high-stakes and low-stakes condition, we restricted comparisons to eventually hired job applicants (*N* = 546) and the sample of job incumbents (*N* = 365). As expected, test-takers in the high-stakes condition had a significantly higher mean person parameter of faking (*M* = 0.07) than test-takers in the low-stakes condition (*M* = −0.77), *t*(909) = 14.81, *p* < .001, *d* = 1.00. Concerning response styles, we expected no mean differences between conditions, and indeed did not find any significant differences for ERS or MRS (see Figure 4A and 4B). Regarding substantive traits, we expected that potential mean differences between conditions would be less pronounced in models accounting for faking than in models ignoring faking. In line with these expectations, there were considerable mean differences between the high-stakes and low-stakes condition when not accounting for faking; however, effect sizes became smaller when adding faking to the model (see Figure 4C–4G).

#### Relationships of Faking With Other Psychological Constructs (Research Question 3)

Exploratively, we investigated relationships of the faking dimension with covariates. As can be seen in Table 4D, estimated latent correlations between faking and the Big Five were all positive, whereas latent correlations between faking and response styles were estimated close to 0. Also, we examined relations of faking with cognitive ability measures available in the dataset. We therefore correlated the estimated person parameters of faking with test-takers’ scores on measures of intelligence, mental speed, and basic arithmetic skills, which yielded significantly positive correlations that were weak to moderate in size (see Table 5). In contrast, relationships of ERS and MRS with cognitive ability measures were consistently negative and smaller in size.

**Table 5.** Empirical Demonstration: Correlations of Big Five, ERS, MRS, and Faking Person Parameters With Cognitive Ability Measures.

	Intelligence	Mental speed	Basic arithmetic skills
E	.09***	.09***	.05**
ES	.18***	.14***	.07***
A	.15***	.15***	.07***
C	.05**	.05**	.00
O	.04*	.03	.00
ERS	−.15***	−.04*	−.06***
MRS	−.14***	−.10***	−.07***
Faking	.21***	.16***	.10***

*Note. N* = 3,046. Person parameters are maximum a-posteriori (MAP) scores. E = Extraversion; ES = Emotional Stability; A = Agreeableness; C = Conscientiousness; O = Openness; ERS = extreme response style; MRS = midscale response style.

*p < .05, **p < .01. ***p < .001

## General Discussion

In this article, we applied multidimensional IRT modeling to account for faking in high-stakes personality assessment data. Specifically, we used a recent parametrization of the MNRM (see Falk & Cai, 2016; Thissen & Cai, 2016) to model faking by means of scoring weights representing each item’s desirability characteristics.

### Summary of Results

The purpose of our simulation was to examine the MNRM approach of modeling faking in terms of the recovery of focal model parameters and model selection. Results showed that accounting for faking can considerably improve parameter recovery when faking is part of the data-generating process. In particular, note the debiasing effect of modeling faking on latent correlations between substantive traits. Models without faking dimension yielded largely positively biased latent correlations, which is consistent with the inflating effect of faking on intercorrelations between scales of a personality inventory (e.g., Ellingson et al., 1999; Klehe et al., 2012; Schmit & Ryan, 1993). The inclusion of a faking dimension in the model, however, debiased estimates and led to a more accurate representation of the true substantive trait intercorrelations. The debiasing effect of modeling faking on latent correlations between substantive traits was also evident in the empirical demonstration.

Crucially, the simulation also showed that modeling faking does not diminish parameter recovery when faking is not part of the data-generating process. For instance, person parameter recovery did not deteriorate when accounting for faking in conditions in which faking was not present in the data. This indicates that a faking dimension does not remove substantive variance from test-takers’ trait scores, which is a major limitation of using SDR scales to account for faking (e.g., de Vries et al., 2014; McCrae & Costa, 1983). At the same time, however, the simulation pointed out that it is essential to make model comparisons and only interpret parameters from a model including faking if this model significantly increases model fit and/or provides a better balance between fit and parsimony over a model ignoring faking. When faking was not present, the MNRM estimated on average non-zero latent correlations between faking and substantive traits as well as non-zero item slopes of faking. Hence, to avoid drawing conclusions from potentially biased parameters in an overparameterized model, researchers and practitioners should always consider model selection criteria before interpreting parameter values. The simulation showed that LR tests, AIC, and BIC can be used for this purpose as they reliably detected overparameterized models, even in samples of *N* = 250. More information on how to deal with the risk of overparameterization and overfitting can be found in Online Supplement II at https://osf.io/f8vgp/.^5^

As is always the case in statistical models, bias and RMSE were higher in smaller samples than in larger samples. To avoid more imprecise parameter estimates than found in this simulation, we advise researchers and practitioners against applying the MNRM in datasets that do not meet the minimum sample size requirements for polytomous and multidimensional IRT models (Dai et al., 2021; de Ayala, 2022). Other than that, it can be informative to look at standard errors and confidence intervals of parameter estimates, which give an indication about the reliability of estimates and constitute a safeguard against overinterpreting unstable point estimates.

Concerning the empirical demonstration, we found that the MNRM approach of modeling faking can also prove successful in real high-stakes assessment data. First, the latent faking dimension explained incremental variance in item responses, which showed in increased model fit and estimated item slopes of faking that were of considerable size (see Online Supplement I for more information on the relative impact of response bias dimensions). Second, divergent validity of the Big Five scales was enhanced by bringing latent correlations closer to values that are more in line with previous research on Big Five intercorrelations (DeYoung, 2006; Digman, 1997). Third, mean differences in substantive trait person parameters between a high-stakes and low-stakes condition (Birkeland et al., 2006; Viswesvaran & Ones, 1999) were reduced. Fourth, faking exhibited considerable relationships with both substantive personality traits and cognitive ability.

### Utility of the Faking Modeling Approach

From a psychometric perspective, the model presented in this article has several appealing features. First, by yielding estimates of substantive trait scores that are adjusted for the influence of faking, the model can afford a purer measurement of the traits of interest compared to models ignoring the response bias of faking. In high-stakes assessments, this helps to ensure that a high faking tendency does not directly lead to more favorable assessment scores, which would otherwise imply a disproportionately elevated chance of being selected for a job, promotion, or the like. Also, it helps to ensure that decision-makers can base their decisions on measures that better reflect the constructs intended to be assessed for the process at hand. Second, the model can debias correlations between substantive traits that are typically inflated through faking. Hence, construct validity in terms of divergent validity is enhanced, which is a desired test feature from a psychometric measurement perspective but is also essential in applied measurement contexts like personnel selection, as it provides practitioners with more nuanced personality profiles of test-takers.

In addition, from a substantive research perspective, modeling faking as in the present article can facilitate the understanding of the substantive nature of the faking construct. The model conceptualizes faking as a continuous interindividual difference variable (cf. Ziegler et al., 2015). Hence, instead of providing only a discrete piece of information about a test-taker’s faking state, the model quantifies the degree of response distortion, which can be used to evaluate the trustworthiness of responses and to study relationships between faking and other psychological constructs. The latter helps to better integrate faking into the nomological network of personality and cognitive ability constructs.

### Advantages Over Other Faking Approaches

Compared to other approaches accounting for SDR and faking, the MNRM approach has important advantages. Whereas classical approaches (e.g., using SDR scales) only afford a separate measurement of SDR or faking and substantive traits, the MNRM approach takes into account the joint influence of substantive traits, response styles, and faking on item responses to disentangle the effects on a latent level. Thus, one can use model-based estimates of substantive trait scores and does not have to rely on a post-hoc control of SDR or faking using, for instance, residuals from a simple linear regression, which holds the risk of removing substantive variance from test-takers’ trait scores (Griffith & Peterson, 2008; Reeder & Ryan, 2011).

Modeling faking by means of the MNRM shares the feature of accounting for test-takers’ faking variation in a model-based manner with other faking modeling approaches. However, it has the crucial extension of accounting for faking effects that are specific to the desirability characteristics of items. A commonly applied latent variable approach to modeling faking is the so-called ideal-employee factor model (e.g., Hendy et al., 2021; Klehe et al., 2012; Schmit & Ryan, 1993), which is essentially a bifactor model where faking represents the general factor and the substantive traits represent the specific factors. This model implicitly assumes that faking is linearly related to response categories for all items. However, if the relationship between response categories and desirability is curvilinear, the model is misspecified. The same criticism can be raised for other recent faking models (e.g., Böckenholt, 2014; Brown & Böckenholt, 2022; Leng et al., 2020; Ziegler & Bühner, 2009). Böckenholt (2014), for instance, developed a three-stage response process model which acknowledges the existence of a response set that is related to item content and refers to a motivation to respond in a way that enhances self-presentation. The model conceptualizes faking as a process of motivated misreporting under which test-takers edit responses by overreporting on desirable items and underreporting on undesirable items. Again, if there are items at which desirability does not increase or decrease monotonically with response categories, the model does not provide a full explanation of the underlying faking process. In contrast, by specifying item desirability characteristics through scoring weights in the MNRM, one can make use of this relevant information in item responses. At the same time, the MNRM allows for correlations between faking and substantive traits as well as between substantive traits themselves, whereas bifactor models of faking, for instance, comprise orthogonal general and specific factors.

Finally, the MNRM approach constitutes a feasible method to account for faking in applied assessment contexts, since the model can be specified and estimated in a straightforward manner on standard computers using open-source software packages for IRT modeling, such as the R package *mirt*. As demonstrated in the simulation, the model also does not require overly large samples. Other modeling approaches of faking (e.g., Böckenholt, 2014; Brown & Böckenholt, 2022; Leng et al., 2020) are considerably more cumbersome to specify, need larger sample sizes, and require knowledge in probabilistic programming languages for Bayesian estimation or commercial statistics software. Furthermore, after having estimated the model in a suitable standardization sample, person parameters for new test-takers can be estimated in only a few seconds, which also facilitates the usability of the model in practice. To guide researchers and practitioners in applying the model, an explanatory syntax file for specifying and estimating the MNRM with item-specific scoring weights in *mirt* can be found at https://osf.io/f8vgp/.

### Limitations and Future Research Directions

Despite promising results in the simulation and empirical demonstration, some limitations of modeling faking by means of the illustrated IRT approach should be mentioned. One limitation concerns the implicit assumption that the same relation between response categories and desirability on a particular item applies to every test-taker. However, if test-takers perceive desirability differently, this assumption can be violated, leading to a potential misalignment between specified and actual scoring weights of faking. Future studies should examine how much consensus in test-takers’ desirability perceptions is necessary such that the presented faking modeling approach still produces satisfactory results. Determining a criterion for the acceptable level of disparity in individual desirability perceptions would be an interesting endeavor for further simulation studies. Also, there can be heterogeneity in how test-takers behave in actual high-stakes situations (e.g., Robie et al., 2007). Some test-takers might indeed try to figure out the most desirable response category at every item and edit responses correspondingly, whereas other test-takers might know how tests are classically scored (i.e., using sum scores) and hence unconditionally choose higher (lower) response categories if they assume that a generally desirable (undesirable) trait is measured. To account for these kinds of heterogeneity in the response process would also be an appealing approach for future model extensions. Relatedly, future research could also try to estimate scoring weights of faking from the data instead of specifying them a priori, which would have the pragmatic advantage of not having to run a pilot study before one can apply the model to empirical data. According to Falk and Cai (2016), the MNRM indeed allows for free estimation of both item slopes and scoring weights, but it remains to be shown that this also works well for the case of faking.

Another challenge of the presented faking modeling approach refers to the fact that, under certain circumstances, scoring weight vectors of substantive traits and faking can exhibit high collinearity. This can make it inherently difficult to disentangle the latent dimensions. The extent of collinearity depends on (a) the variability of desirability trajectories across items and (b) the number of substantive traits modeled. In the extreme case, namely if all items had desirability trajectories that were linearly increasing in the direction of the substantive trait and if only one substantive trait was modeled, faking and the substantive trait dimension would be redundant and thus not separable. One can argue that disentangling faking from substantive traits will be facilitated with more items exhibiting nonmonotonically increasing, inverted-U-shaped, or even decreasing desirability trajectories, as well as with more substantive trait dimensions being modeled, since this will reduce the general overlap of scoring weight vectors. Our simulation featured a scenario with five substantive traits where the majority of items had monotonically or nonmonotonically increasing desirability trajectories and some had inverted-U-shaped trajectories, which is representative of the personality test from our empirical demonstration. To address the question of how much collinearity between scoring weight vectors is acceptable for a proper separation of substantive traits and faking, further studies are needed that go beyond the scope of this article.

Finally, it would also be worthwhile to study the empirical implications of the model’s adjustments of substantive trait scores in more detail. Despite encouraging findings in the simulation and empirical demonstration of this article, future studies are required to fully answer the question of whether the substantive trait score adjustments afforded by the model indeed lead to a better representation of test-takers’ substantive trait levels. For such an investigation, data situations would be appealing in which the same test-takers provide real high-stakes data along with personality measures that are less susceptible to faking, such as multidimensional forced-choice (MFC) measures (e.g., Brown & Maydeu-Olivares, 2013; Cao & Drasgow, 2019) or observer ratings of personality (e.g., Connolly et al., 2007; Oh et al., 2011).

### Conclusion

To conclude, the MNRM provides an appealing framework for modeling faking in high-stakes personality assessments. Specifying scoring weights according to a-priori information about social desirability enables researchers and practitioners to model item-specific effect patterns of faking. While the simulation in this article found good parameter recovery and precise model selection under different conditions, the empirical demonstration showed that it is worthwhile to model faking in real high-stakes assessment data. We hope to stimulate future research on the model of this article or related models accounting for response tendencies that manifest idiosyncratically depending on item content and assessment context. Continued research in this area will be fruitful in deepening the understanding of how response biases affect self-report measures and will help to further improve the measurement of substantive personality traits in special assessment contexts like high-stakes settings.

## Notes

1. Note that approaches seeking to prevent faking in the first place, such as the multidimensional forced-choice format (MFC; e.g., Brown & Maydeu-Olivares, 2013) or the use of items that are neutral in terms of social desirability (e.g., Bäckström et al., 2009), yield estimates of substantive trait scores that are assumed to not be confounded with faking but do not readily provide an estimate of each test-taker’s faking degree. Hence, such approaches are not suitable to study the substantive nature of faking.

2. Note, however, that the described model represents a dominance IRT model as opposed to an ideal-point IRT model. Dominance models are models in which the probability of choosing response categories with higher scoring weights increases monotonically with higher person parameters. In contrast, ideal-point models assume that persons more strongly endorse items the closer their trait level is to the item’s location on the trait continuum (e.g., Chernyshenko et al., 2007; Tay & Ng, 2018). That is, the probability of an item response in ideal-point models is determined by the distance between person and item parameters, which implies that intermediate trait levels can be associated with higher probabilities of high rating scale categories. This is reflected in a nonmonotonic item response function (IRF), which maps trait levels onto the expected item response. IRFs are to be distinguished from trajectories between response categories and social desirability, which depict how rating scale categories are related to desirability at a specific item. Since the MNRM is a dominance model, higher faking levels are always associated with higher probabilities of choosing more desirable response categories, though these categories may not necessarily be the highest categories on the rating scale.

3. In the simulation, scoring weights of all latent dimensions were linearly transformed to a common range to achieve a comparable metric of scoring weights. Such transformations facilitate the interpretation of item slopes and do not impact model fit or the estimation of latent correlations and person parameters (cf. Falk & Ju, 2020, for details).

4. These population values were chosen to simulate item response distributions covering all response categories in the present parameter constellation.

5. In this Online Supplement, we report a small simulation investigating the effect of modeling faking on out-of-sample predictive accuracy, which refers to the ability of a model to make precise predictions of new datapoints. Results indicated that accounting for faking improves predictive accuracy when faking is present in the data, and, crucially, does not deteriorate predictive accuracy when faking is absent (i.e., when the model is overparameterized).
